# Towards a scalable and accurate quantum approach for describing vibrations of molecule–metal interfaces

**DOI:** 10.3762/bjnano.2.48

**Published:** 2011-08-10

**Authors:** David M Benoit, Bruno Madebene, Inga Ulusoy, Luis Mancera, Yohann Scribano, Sergey Chulkov

**Affiliations:** 1 Nachwuchsgruppe Theorie – SFB 569, Albert-Einstein-Allee 11, University of Ulm, D-89081 Ulm, Germany; 2 Department of Chemistry, The University of Hull, Cottingham Road, Kingston upon Hull HU6 7RX, United Kingdom; 3 L.A.D.I.R Boite 49, Bat F74, Université Pierre et Marie Curie, 4 Place Jussieu, F-75252 Paris, France; 4 Technische Universität München, Theoretical Chemistry, Lichtenbergstr. 4, D-85747 Garching, Germany; 5 Theoretical Chemistry, Albert-Einstein-Allee 11, University of Ulm, D-89081 Ulm, Germany; 6 Laboratoire Interdisciplinaire Carnot de Bourgogne,UMR 5209 CNRS – Université de Bourgogne, 9 av. A. Savary, BP 47870, F-21078 Dijon Cedex, France

**Keywords:** computational scaling, grid computing, molecule–metal interactions, periodic density functional theory, vibrational theory

## Abstract

We present a theoretical framework for the computation of anharmonic vibrational frequencies for large systems, with a particular focus on determining adsorbate frequencies from first principles. We give a detailed account of our local implementation of the vibrational self-consistent field approach and its correlation corrections. We show that our approach is both robust, accurate and can be easily deployed on computational grids in order to provide an efficient computational tool. We also present results on the vibrational spectrum of hydrogen fluoride on pyrene, on the thiophene molecule in the gas phase, and on small neutral gold clusters.

## Introduction

The study of molecular vibrations has been a topic of continued interest in chemistry for over a century, starting with the work of William Coblenz in 1905. The main driving force behind these studies has been the correlation that exists between molecular structure and observed vibrational transition frequencies. Thus, a measured vibrational frequency depends directly on the strength of the bonds present in the molecule and on the mass of its atoms. Moreover, particular bonding patterns can give rise to specific collective motions of parts of the molecule; the corresponding oscillation frequencies of this set of atoms are usually known as group frequencies and are characteristic of a particular structural motif.

In heterogeneous catalysis, for example, the shift of the adsorbate vibrational frequencies allows us to follow the progress of surface reactions and provides important information on the bond strength and location of the adsorbate. A typical example is carbon monoxide, which can be used as a surface probe, as the C=O stretch frequency is very sensitive to the adsorption site of the molecule. This property was identified very early on, and was used by Yang and Garland [1] to investigate, for example, the binding mode of a CO molecule deposited on supported rhodium films and to suggest possible mechanisms for hydrogenation and oxidation reactions on this type of metal surface.

A recent development for vibrational spectroscopy in the field of surface characterisation is the use of scanning tunnelling microscopy (STM) to record single-molecule spectra. This relies on the technique of inelastic electron tunnelling spectroscopy (IETS), developed in the mid-1960s [2], and performs measurements on a single molecule using an STM tip as a contact instead of a macroscopic metal/oxide layer. There are a number of studies (see [3] for a review) that have shown the flexibility of the combination of STM with IETS for the investigation of single adsorbates on the nanoscale. Recently, our group was able to demonstrate that, with an appropriate anharmonic model of the vibrational structure, STM–IETS can be used to determine the adsorption geometry of a 4-mercaptopyridine molecule on the Au(111) surface [4]. This study confirmed that a coordination of the sulfur atoms by two gold atoms (bridge site or defect site) is likely to be the preferred binding mode for the adsorbate. This is in agreement with other theoretical predictions [5].

This paper describes our efforts over the past few years in developing scalable techniques for the computation of anharmonic vibrational frequencies of large systems, with a particular focus on the interface between organic molecules and metal surfaces. Such systems are of significant interest in the field of nanotechnology as they are the building blocks of functionalised interfaces and are also related to catalysis processes. The interface between the molecular world and the condensed phase is still relatively poorly understood, and the vibrations of adsorbed molecules provide us with information on their surface binding strength and on the dynamic processes that occur at the interface.

Our aim is to further the understanding of adsorbate vibrations by developing a set of techniques that render their accurate prediction possible at modest computational cost. We show that a systematic approach to the description of anharmonic vibrational structure, based on the vibrational self-consistent field (VSCF) method and its correlated variants, combined with an accurate yet scalable description of the underlying potential energy landscape, leads to a flexible way of describing these systems.

This paper is organised as follows: First, we give a brief introduction to the global theoretical framework used for our implementation. We then describe the details of our local implementation of the direct-VSCF technique, followed by a detailed account of our efforts to speed up vibrational correlation corrections for large systems. Next, we introduce a distributed approach for the computation of the necessary potential energy surfaces (PES). We finish the paper with two new developments: The frequencies of the thiophene molecule are calculated from a PES constructed with periodic density functional theory (DFT), and an assessment of DFT for the description of properties of small neutral gold clusters is given. We conclude this paper with an outlook on future work in the field of theoretical descriptions of vibrational spectra of adsorbed molecules.

## Results and Discussion

### Theoretical framework

The theoretical description of molecular vibrations is remarkably simple. In its most basic formulation, the harmonic approximation can successfully describe a very large number of observations. This has been shown in the excellent monograph by Wilson Jr., Decius and Cross [6], in which they demonstrated a straightforward procedure to obtain harmonic vibrational frequencies for any given molecule. The success enjoyed by the harmonic approximation over the years, combined with its relative simplicity, has made it a de facto standard for the prediction of vibrational frequencies.

From a theoretical point of view, the accuracy of the data obtained from high resolution spectra provides an ideal opportunity to validate the interaction models used to describe the observed molecule, be they empirical force fields or quantum chemical Hamiltonians, as vibrational spectra probe bond strengths directly. Unfortunately, standard vibrational theory is often powerless to explain the subtle changes observed in the vibrational spectrum when a molecule undergoes a conformational change or is adsorbed on a surface. Indeed, the harmonic approximation can only go so far, as it mainly considers infinitesimal displacements from the equilibrium structure and does not truly explore the local energy landscape of a flexible molecule. This approximation usually results in a severe overestimation of experimental frequencies by up to 200–300 cm^−1^ for single stretching frequencies.

As an alternative to the harmonic approximation, two methods for computing anharmonic spectra are of particular interest: Molecular dynamics and the vibrational self-consistent field (VSCF) approach. Both techniques can go beyond the harmonic approximation, and provide a solid basis for the interpretation of complex vibrational experiments.

The first method offers a fully classical approach to anharmonic corrections that can easily include solvation (or more generally environment effects) and finite temperature effects. This latter aspect can be important in the case of conformationally flexible molecules. Due to its conceptual simplicity and the ready availability of reliable empirical force fields (or forces computed ab initio), molecular dynamics is currently the most popular method for determining anharmonic frequencies of large systems (see [7] for an overview of some applications for biological systems). However, given its classical nature, this technique cannot account consistently for quantisation effects such as Fermi resonances, overtones or combined excitations.

In contrast, VSCF-based methods provide a fully quantum mechanical picture of anharmonicity in molecular systems and, as such, are able to account for resonance phenomena, combination bands and vibrational overtones in a hierarchical and consistent manner. While temperature effects can be included using a statistical mechanics framework, these are usually neglected, and thus the approach is better suited to the description of low temperature vibrational spectra, such as those obtained in supersonic jet expansions or in ultrahigh-vacuum environments.

In most implementations of VSCF-based approaches, the required resolution of the vibrational Schrödinger equation means that it remains a time-consuming process. This is due to the necessity to first construct a potential energy surface (PES), which is then used to compute the vibrational Hamiltonian. The PES can also be generated directly from ab initio programs (direct-VSCF), thus leading to a straightforward route from electronic structure theory to a measurable vibrational spectrum, within the Born–Oppenheimer approximation.

This paper focuses on VSCF-based techniques and describes our local implementation, PVSCF, that aims to reduce the computational cost of the direct-VSCF method for large and interfacial systems. In order to render this approach suitable for large systems, two main aspects of the technique need to be considered: The generation of the PES and the vibrational computation itself.

### Expressing the potential energy surface

In order to obtain a manageable and compact description of the multi-dimensional PES for large systems, it is desirable to use an approach that has a physical underpinning and whose accuracy can be improved systematically. Rabitz et al. [8–9] showed that a many-body decomposition leads to a convenient hierarchical representation of the PES. In their approach, the PES is expanded in a series of one-dimensional terms, two-dimensional couplings, three-dimensional couplings, etc. This expansion guarantees convergence, as a 3*N_a_* − 6 dimensional expansion describes the complete PES for a problem consisting of *N_a_* atoms in the case of an isolated molecule. Note that other representations, such as polynomial expansions, do not necessarily possess such well defined multi-dimensional convergence properties. For large systems, Gerber et al. [10] have shown that a truncation of the expansion to two-dimensional (2-D) couplings built on the normal modes of the system is sufficiently accurate for most applications. This considerably reduces the computational effort of generating PES for the description of vibrational properties of large systems. Another advantage (which will become clearer later) of this type of representation is that it is isomorphic with electronic structure theory, where all interactions are two-body interactions. The direct method suggested by Gerber et al. [10] reduces the computation of the global PES for a given system to that of a local part of the energy landscape located around the energy minimum and expanded in a series of 1-D and 2-D cuts, calculated point-by-point through ab initio techniques. Thus, in this method, the speed at which the PES can be computed is directly related to the number of mode–mode couplings taken into account and to the amount of computing power allocated to the ab initio calculation of each grid point. However, the latter constitutes one of the main computational bottlenecks of the technique for large systems.

### Accelerating anharmonic vibrational calculations

Several years ago [11], we formulated the concept of the fast-VSCF technique and demonstrated that the computationally expensive stages of direct-VSCF calculations, namely the computation of mode–mode coupling potentials using ab initio techniques, can be efficiently pre-screened using fast and approximate electronic structure methods. Indeed, for any given molecular system, we showed that the coupling pattern is mainly defined by the chemical nature of the molecule and the choice of coordinate system for the normal-mode expansion (see [12] for details of the influence of coordinate systems on VSCF calculations). Thus, any level of electronic structure theory can be used to obtain a qualitative assessment of the strength of mode–mode coupling, as long as this level offers a reasonable description of the potential energy surface. This concept opens the way to a two-level approach to the determination of couplings that we have called the fast-VSCF method. Other groups have further developed such a multi-level approach in order to compute different parts of the PES through a set of electronic structure calculations of decreasing accuracy [13–14] and have achieved reasonable accuracy for small to medium-sized molecules.

The fast-VSCF technique uses the fact that there are preferred channels along which vibrational modes interact with each other and that these channels dominate the vibrational dynamics of the system. This is particularly manifest when a molecule contains similar functional groups, such as C–H bonds or C=O groups, that necessarily interact with one another for symmetry reasons (vibrations of identical functional groups should couple together even if it is only through Fermi resonances). Once these channels have been identified, it is possible to construct an approximate representation of the vibrational Hamiltonian that contains only these important interactions, and the resulting reduced-dimension model leads to a very reasonable description of the molecular vibrations. This approach bears similarities to some of the semi-empirical techniques used in electronic structure theory, such as the complete neglect of differential overlap (CNDO) approach of Pople et al. [15], but it mainly originates from considerations of the usage of locality in large-scale electronic structure methods (so called order-N approaches). Indeed, the idea of restricting the calculation to a local environment (often defined as nearest-neighbour interactions) is the major tenet of a large number of linear-scaling electronic structure methodologies, and in fast-VSCF we limit the treatment of vibrational correlation to a “local” group of modes. The delocalised nature of rectilinear normal modes usually renders a direct prediction of nearest-neighbour interactions difficult. There have been attempts at using criteria based on localisation techniques to determine the importance of a given mode–mode pair using only normal modes [16]. This approach relies on the assumption that two strongly coupled modes are likely to move the same atoms while two weakly coupled ones are not, and has so far shown moderate success at predicting all strong couplings of the systems investigated.

Unfortunately, generic non-trivial rules for determining the preferred channels of a given molecule have so far proved elusive, but we are pursuing research in this direction. Thus, for a practical implementation, the fast-VSCF approach usually requires an initial qualitative exploration of the PES of the system, albeit at a low level of theory.

By greatly reducing the time spent on the PES calculation, the fast-VSCF approach enables the investigation of larger systems, which would have otherwise been out of the reach of a standard direct-VSCF calculation. In particular, we have shown [11] that correlation-corrected fast-VSCF (fast-VMP2) achieves increases in speed of up to 80 times that of standard direct-VMP2 calculations on the systems studied.

Fast-VSCF is a technique that was initially built to accelerate PES calculations, but it also confers a particular structure on the vibrational Hamiltonian (rendering it sparser) that can be further exploited in the vibrational correlation treatment to construct efficient algorithms, as will be shown later in the paper.

### Our local VSCF implementation

Our aim is to develop a program to compute accurate vibrational frequencies of polyatomic system in the gas phase, condensed phase and at interfaces. We start by assuming that the Born–Oppenheimer approximation is valid, and thus we first need to compute the PES of the system and then we consider the nuclear motion by solving the vibrational Schrödinger equation on the PES obtained.

Our implementation choices have been guided by the following requirements:

- The program should be an external code that could be interfaced with any given ab initio electronic structure code to ensure maximum flexibility during the construction of the PES. This enables us to change ab initio package according to the availability of electronic structures methods or performance of the implemented algorithms. This capability is a necessity for the investigation of large systems as the many packages that implement efficient density functional theory (DFT) are still in development and change on a regular basis.
- Scalability of the vibrational approach means that the program should be able to run on many cores (i.e., parallel code). Computationally speaking, the PES construction remains the most demanding part of the calculation as we typically need on the order of 10^6^ points for large systems. However, this is a highly parallel task given that the various grid points are practically independent and we will discuss our strategy for scalable PES computation later in the paper. Finally, once the PES is computed, the vibrational treatment can become the limiting factor for systems containing a large number of modes. Thus there is a necessity for the development a vibrational treatment that may be parallelized for such cases.
- Ideally, the program should use the ab initio energy grid points themselves and avoid fitting the PES to a functional form. This ensures that the accuracy of the PES is preserved throughout the calculation.
- Finally, not only do we need the energy of the vibrational ground state of the system but also that of its vibrationally excited states in order to compute fundamental frequencies and overtones or combination bands. We also require a representation of the vibrational wave function in order to be able to compute vibrationally averaged geometries for a given state or in order to compute infrared transition intensities.

The core part of our program is built around a solver for 1-D radial vibrational Schrödinger equations of the kind:

[1]
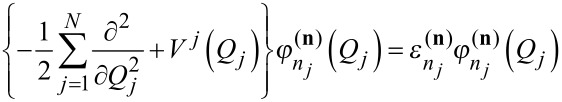


where *Q_j_* is usually a mass-weighted normal coordinate of the system, *V ^j^*(*Q_j_*) is a generic 1-D potential, *V ^j^*(*Q_j_*) is the single-mode wave function, 
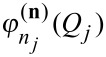
 the corresponding eigenenergy and (**n**) = {ν_1_,ν_2_,…,ν*_n_*} represents the excitation quanta for each vibrational mode. This routine must be fast, accurate and flexible enough to treat any kind of 1-D potential. After evaluating a number of approaches based on different basis sets for construction of the Hamiltonian representation (particle-in-a-box eigenfunctions, gaussian functions, harmonic oscillator eigenfunctions, delta functions), we solve Equation 1 using the Fourier grid Hamiltonian (FGH) approach proposed by Balint-Kurti and Martson [17–18]. This approach is fast and robust enough to accommodate any kind of potential that is likely to occur during the vibrational calculation (potential with one or several minima, periodic or aperiodic potential), and it gives the eigenvalues and the eigenfunctions as the results. The wave functions obtained are also easily integrable, as they are based on delta functions. The limiting part of the calculation is the diagonalisation of an *N_g_* × *N_g_* matrix, where *N_g_* is the number of grid points (which must be even in this approach). We use mathematical libraries (Lapack) to ensure the scalability of the approach.

There are two possible points of failure in our technique, one is using too few or an odd number of grid points and the other is an insufficient exploration of the 1-D potential. The number of points, *N_g_*, can be fixed by the user in our implementation but the default value of *N_g_* = 128 is usually sufficient for most applications, and convergence with respect to *N_g_* is easily checked. This leads to a relatively small computational burden on the diagonalisation routines. As the potential energy is usually computed ab initio on a grid that is sparser than *N_g_*, the 1-D PES is interpolated using a cubic spline procedure (Numerical Recipes [19], modified by M. Lewerenz) in order to obtain the values on the *N_g_* grid points. By default, the limits of the interpolated grid are those of the 1-D ab initio PES grid. The issue of insufficient exploration of the potential is more difficult to resolve, but we have implemented additional options to check the validity of the PES range: Basic linear extrapolation using energies derived from the limits of the potential, or usage of an infinite wall potential at the limits. In practice, if the first seven eigenvalues and wave functions do not change when extrapolating the potential, the initial potential range was large enough.

#### Diagonal (1-D) calculation

**Figure 1:**
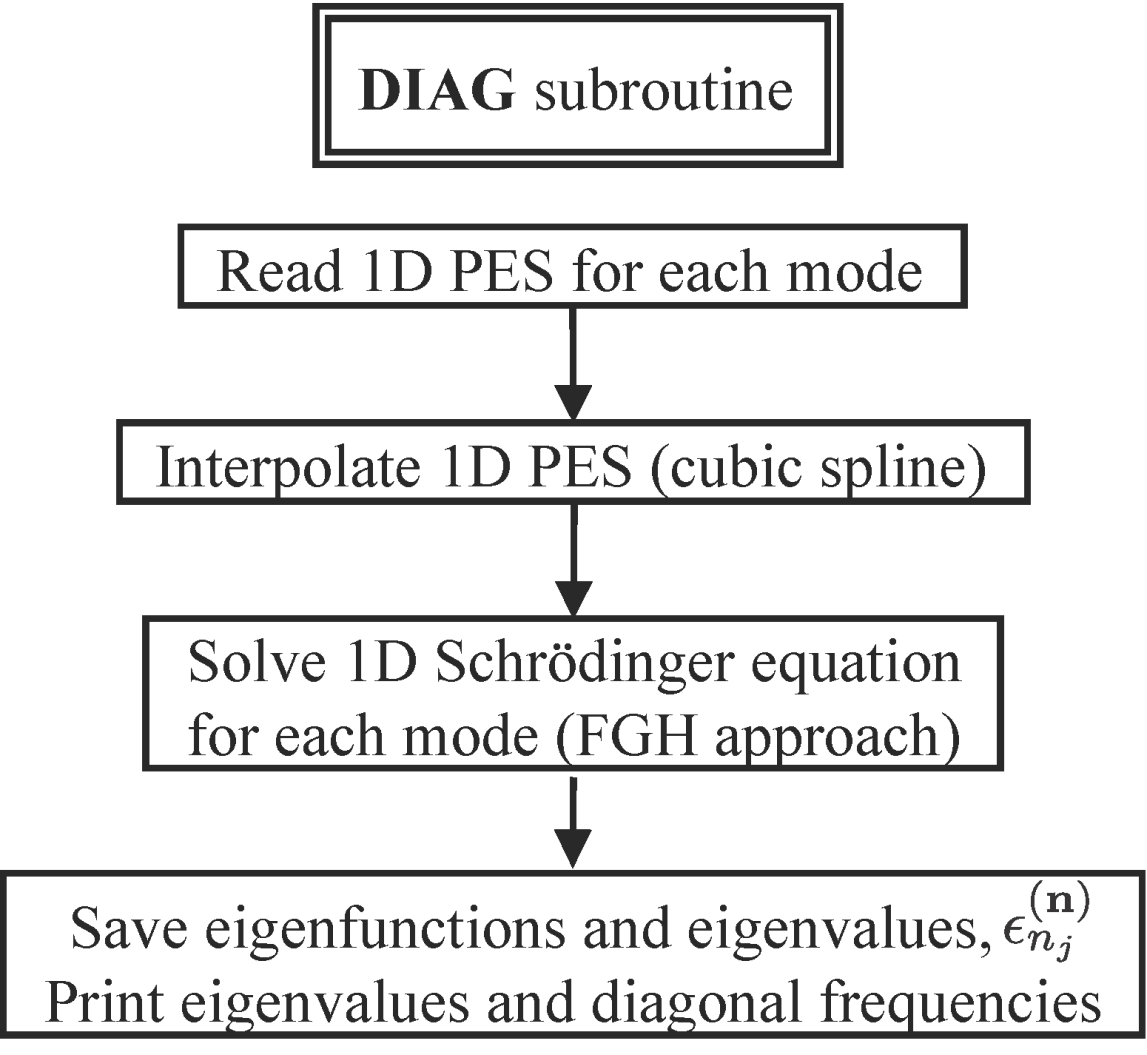
Flow-chart diagram of the computer code used to compute diagonal frequencies.

This is the first step beyond the harmonic approximation and a flow-chart diagram of our routine is shown in Figure 1. In this part, the 1-D vibrational Schrödinger equation for each mode is solved with the FGH subroutine and the potential obtained from 1-D PES cuts along each mode. The anharmonic frequencies are then obtained by simply subtracting the ground state energy from the first excited state energy for each vibrational mode. This leads to improved vibrational frequencies compared to the harmonic approximation, but even though these diagonal frequencies reflect the main anharmonic contributions, they do not include any vibrational correlation between modes. For many-mode systems, the diagonal approximation is not sufficient to obtain realistic vibrational frequencies but the solutions obtained at this stage can be improved through the VSCF procedure outlined below.

#### VSCF procedure

The main idea is to use an effective mean-field 1-D potential, 
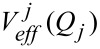
, along each mode, instead of the diagonal potential, in order to take into account the vibrational coupling between modes:

[2]
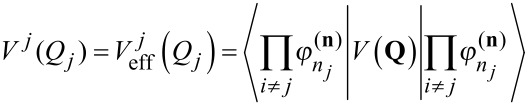


where *V*(**Q**) is the full potential energy surface of the *N*-dimensional problem and contains all the coupling terms of the potential energy operator. The main computational difficulty in solving Equation 1 at this stage comes from the evaluation of the multi-dimensional integral needed to compute the mean-field potential. To overcome this difficulty, Jung and Gerber [20] suggested using the following *n*-body representation of the potential energy surface:

[3]
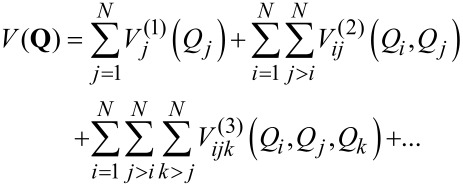


For rectilinear normal-mode coordinates, Chaban et al. [10] have shown that a pairwise approximation (i.e., including up to 
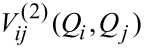
) for *V*(**Q**) is sufficient to give reasonable vibrational frequencies for large molecular systems. This does not imply that higher-order couplings (three or more mode couplings) can always be safely ignored, as Bounouar and Scheurer [21] have shown that neglecting 3-D couplings can lead to artefacts for some systems. In such cases, an internal or (more generally) a curvilinear coordinate representation of the PES leads to a much more accurate description of mode–mode couplings (see [21] and [12], for example), and the error introduced by neglecting 3-D couplings and above can thus be strongly reduced. Yet the a priori definition of a suitably de-coupled set of coordinates for periodic systems remains a complex issue. We are currently investigating optimum decoupling techniques for such systems in our laboratory. In the present study, however, we use a rectilinear 2-mode representation of the PES, despite its limitations, as this simplifies the calculation of the matrix elements involving the potential operator, such that they require only two-dimensional integrals at most. Moreover, in order to speed up the calculations on parallel computers, we make use of the OpenMP library to distribute the integral calculations to many processors.

For a given vibrational state (**n**) = {ν_1_,ν_2_,…,ν*_n_*}, we can compute an initial 
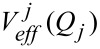
 for each mode *j*, using the vibrational wave functions obtained from the diagonal calculations subroutine and the 2-D PES cut. This effective potential is then used to solve the 1-D Schrödinger equation for each mode. This leads to new eigenvalues and eigenfunctions that can be used to build the state wave function. Note that, in the VSCF approach, we use a separable product of normal coordinate functions to represent the total wave function:

[4]
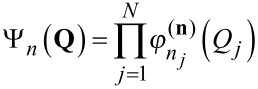


We can then use the new state wave function to compute a new 
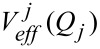
 for each mode, and solve again the 1-D Schrödinger equation for each mode. This sequence of operations is performed until convergence of the total SCF energy:

[5]
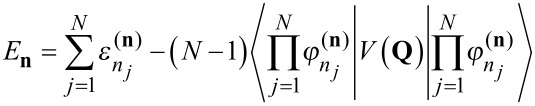


**Figure 2:**
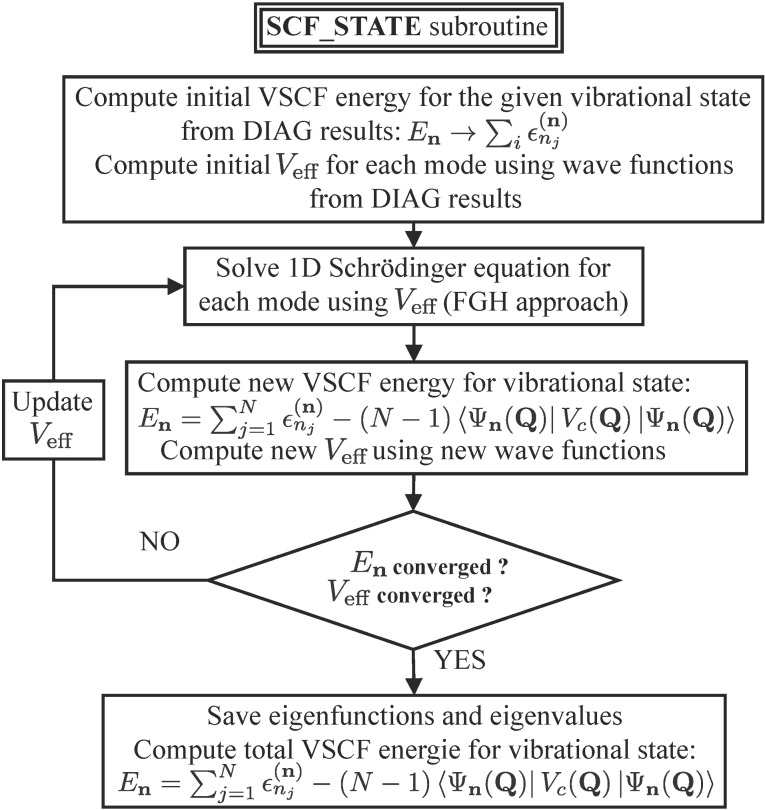
Flow-chart diagram of the computer code used to compute the VSCF state energy.

In order to ensure a stable wave function for the subsequent correlation treatment, we also monitor convergence of the effective potential. Our implementation of the VSCF procedure for a given state is shown in Figure 2.

Although the convergence of the VSCF procedure is usually surprisingly smooth, in some cases convergence problems can occur. These often originate from the use of an inadequate coordinate system (e.g., rectilinear coordinates to describe a torsional motion, see also [12]), or from the limitation of the PES expansion to second order. In order to try and remedy convergence problems when a change of coordinate or a higher-order expansion are not possible, we implemented the following two techniques in our code:

- One possibility is to slow down the change in the effective potential by using a density mixing scheme. Compute the new effective potential by adding some of the effective potential obtained during the previous iteration, such as Equation 6, where *b* is a real number between 0 and 1, given by the user.

[6]
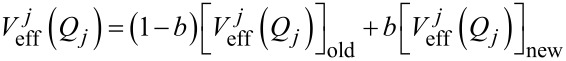

- The second option is to scale down the second-order terms of the PES. This automatically decreases the coupling between modes and usually improves convergence. Such a scaling procedure is also implemented in the VSCF part of the GAMESS-US [22] suite of ab inito programs. One advantage of the technique is that it is known to converge in the limit of zero scaling factor (diagonal situation) and the coupling can be “switched on” progressively to include more coupling. However, this approach must be used with caution, as it changes the potential energy surface to force convergence, and, without further corrections at the correlated level of theory, leads to an inadequate result for the VSCF approach when compared to the fully coupled system.

**Figure 3:**
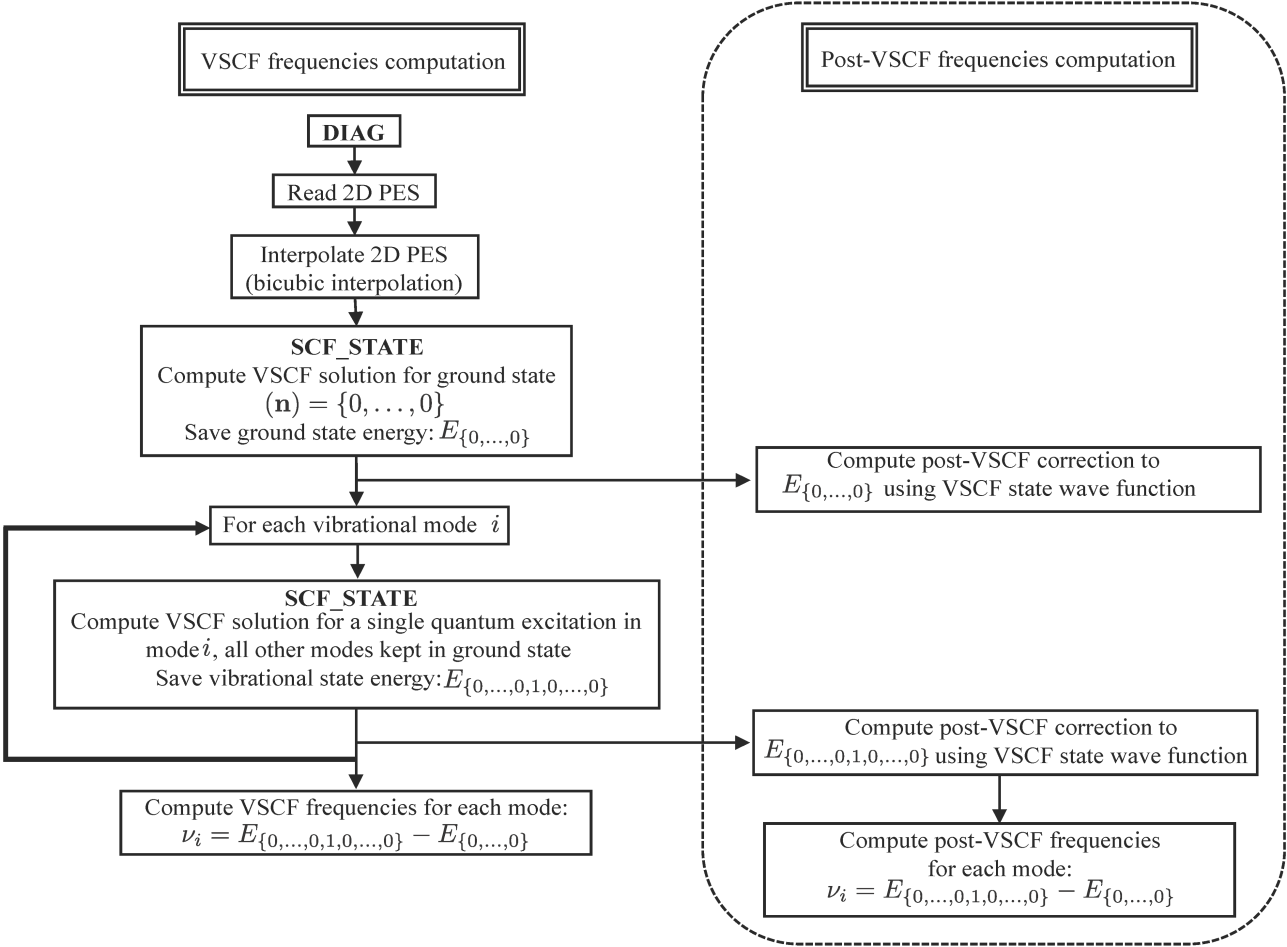
Flow-chart diagram of the computer code used to compute the VSCF and post-VSCF frequencies (dotted part of the diagram).

Finally, the main algorithm used to compute VSCF frequencies is shown in Figure 3. Once the diagonal solutions have been computed, the program reads in the 2-D PES cuts and interpolates them using a bicubic interpolation algorithm [23]. The VSCF energy is then computed first for the ground state {0,…,0} and then for all necessary singly excited vibrational states, {0,…,0,1,0,…, 0}. The anharmonic frequencies are finally computed by a simple subtraction of the relevant vibrational state VSCF energies:

[7]
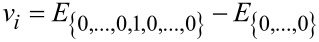


Note that all state energies can be corrected (right part of the flow chart) to include vibrational correlation using a range of post-VSCF methods described in the next section, and the corresponding corrected frequencies are simply calculated in the same way as Equation 7.

### Improvement of the vibrational mean field approach

#### Vibrational Configuration Interaction: The VSCF/VCI approach

The vibrational mean field scheme is well adapted if the intermodal coupling potential is very weak. However, in most cases this condition is not fulfilled and the results of the SCF approach need correcting for mode–mode interactions. There are several ways to include vibrational correlation contribution, and, due to the analogy between VSCF and standard electronic structure theory, these corrections are usually expressed in a formalism based on the electronic Hartree–Fock method. Over the years, a number of groups have developed correction techniques such as the Vibrational Coupled Cluster (VCC) [24–26], Vibrational Multi-configurational SCF (VMCSCF) [27], Vibrational Mean Field Configuration Interaction (VMFCI [28–29]), Vibrational Configuration Interaction (VCI) [30–32] or the MP2-based methods [33–34], to name a few. This last set of approaches is very attractive for large systems as perturbative techniques usually do not require the solution of an eigenvalue problem. The most common perturbation correction is based on the Møller–Plesset formalism and is usually limited to second order of theory (although the use of higher orders has been carefully examined by Christiansen [24]). However, this approach is explicitly pertubative and thus assumes that the intermodal coupling is weak. This limitation has motivated the development of other methods. In our computer code PVSCF, we have mainly implemented the VMP2 (perturbative) and the vibrational configuration interaction scheme VCI (variational method). These two methods use the results of a preliminary VSCF calculation to compute the correlation correction. In this section, we will focus on the variational approach. The principal task of this approach is to diagonalise the following Hamiltonian:

[8]
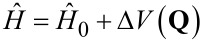


where *Ĥ*_0_ is the vibrational mean field Hamiltonian computed in the VSCF step and Δ*V* the difference between the true *N*-dimensional potential operator (in our case limited to 2-mode representation (2MR) contributions) and the full mean field potential operator,

[9]
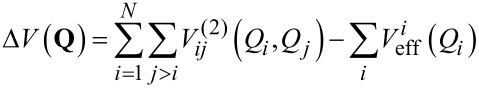


Our implementation in PVSCF is made in a very flexible way. Before giving more details we will present the principal idea of this technique. Since the VSCF configuration is not sufficient to reproduce the anharmonic coupling between modes, we build a virtual basis set that can be used to express the full Hamiltonian by exciting the mean field modal basis. This provides a large number of virtual configurations based on the initial VSCF state, and the Hamiltonian is then diagonalised in this virtual basis (VCI basis). The initial VSCF step can be considered as a preconditioning scheme and gives a more physical vibrational basis than does a basis of uncoupled harmonic oscillators. The size of the virtual configuration basis set 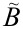 can be controlled by a threshold parameter that limits the excitation of each quanta and/or the sum of quanta over all vibrational modes. The term “virtual” indicates that all excited modal wave functions were computed from a single reference configuration, optimised with the VSCF technique. If the size of the VCI basis is reasonable (size up to around 10,000 elements), the Hamiltonian matrix can be directly diagonalised using routines from standard scientific libraries (e.g., Lapack). Nevertheless, despite the use of thresholds in order to reduce the number of virtual configurations, the VCI basis can very easily grow to a significant size for large systems and become impossible to diagonalise without further treatment.

As our implementation is aimed at large molecular and interfacial systems, we have then implemented an iterative diagonalisation solver based on the Davidson algorithm [35–37], which is well adapted when we have a good zeroth-order hamiltonian (in our case the mean field one). Moreover, as we are usually only interested in the bottom part of the vibrational spectrum, a full diagonalisation of the Hamiltonian matrix is not necessary. This method was firstly applied to this type of vibrational problem by Handy and coworkers [38] and is very well adapted for sparse matrices. The principle of the Davidson algorithm is presented below in Table 1.

**Table 1:** Davidson scheme used to converge a specific state 
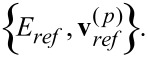
 The 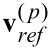 eigenvector is expressed as the VSCF reference state plus the contribution of the excited virtual configurations.

(0) Initialisation (*p* = 1)	Define an initial vector **u**_0_ as the VSCF reference state.
(1) Diagonalisation	Diagonalise **H** in the {**u**_0_, …, **u***_p_*_−1_} basis set 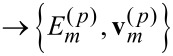 solutions
(2) Convergence	Form residual 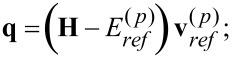 if |**q**| ≤ ε exit.
(3) Preconditioning	Precondition with the zero-order VSCF Hamiltonian **H**_0_ 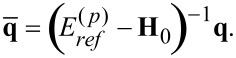
(4) Orthonormalisation	Define a new vector **u***_p_* orthogonal to the previous ones 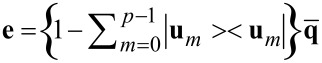 and normalize **u***_p_* = **e**/|**e**|.
(5) Loop *p* := *p* + 1, back to (1)	

### Extension of VSCF/VCI for large systems

#### Direct Davidson diagonalisation

An initial strategy for large systems is to use the Davidson algorithm and store the pre-computed Hamiltonian matrix, expressed in the virtual configuration basis, in the main memory. The action of the Hamiltonian is then obtained through a matrix vector product **H** · **v** where the size of the matrix is *M* × *M*, where *M* is the length of the initial seed vector **v**. However, storing the full Hamiltonian in the main memory can be prohibitive and is not always possible in practice when we consider systems of several tens of modes. For these situations, we have the option of computing the action of the Hamiltonian on the seed vector on the fly, and each matrix element *H_ij_* is then computed only when necessary, thus avoiding large memory requirements. In practice, this approach is suitable for large systems and circumvents the problems of memory requirements but at the detriment of computational speed.

#### A reduced-coupling approach for large mode systems

A different technique is implemented in PVSCF for the evaluation of the potential matrix elements of the VCI matrix. In order to reduce the timing cost of those elements, we use a number of reduced-coupling approaches. One of them is the single-to-all (STA) approach which leads to a significantly reduced computational scaling for large systems [39–40]. Indeed, only the two-mode potential coupling terms involving active modes (*N_Ã_*) with themselves or with inactive ones (*N_Ĩ_*) are used and thus the number of necessary terms in two-mode representation of the potential is highly reduced,

[10]
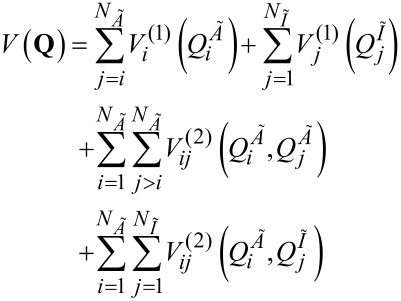


**Figure 4:**
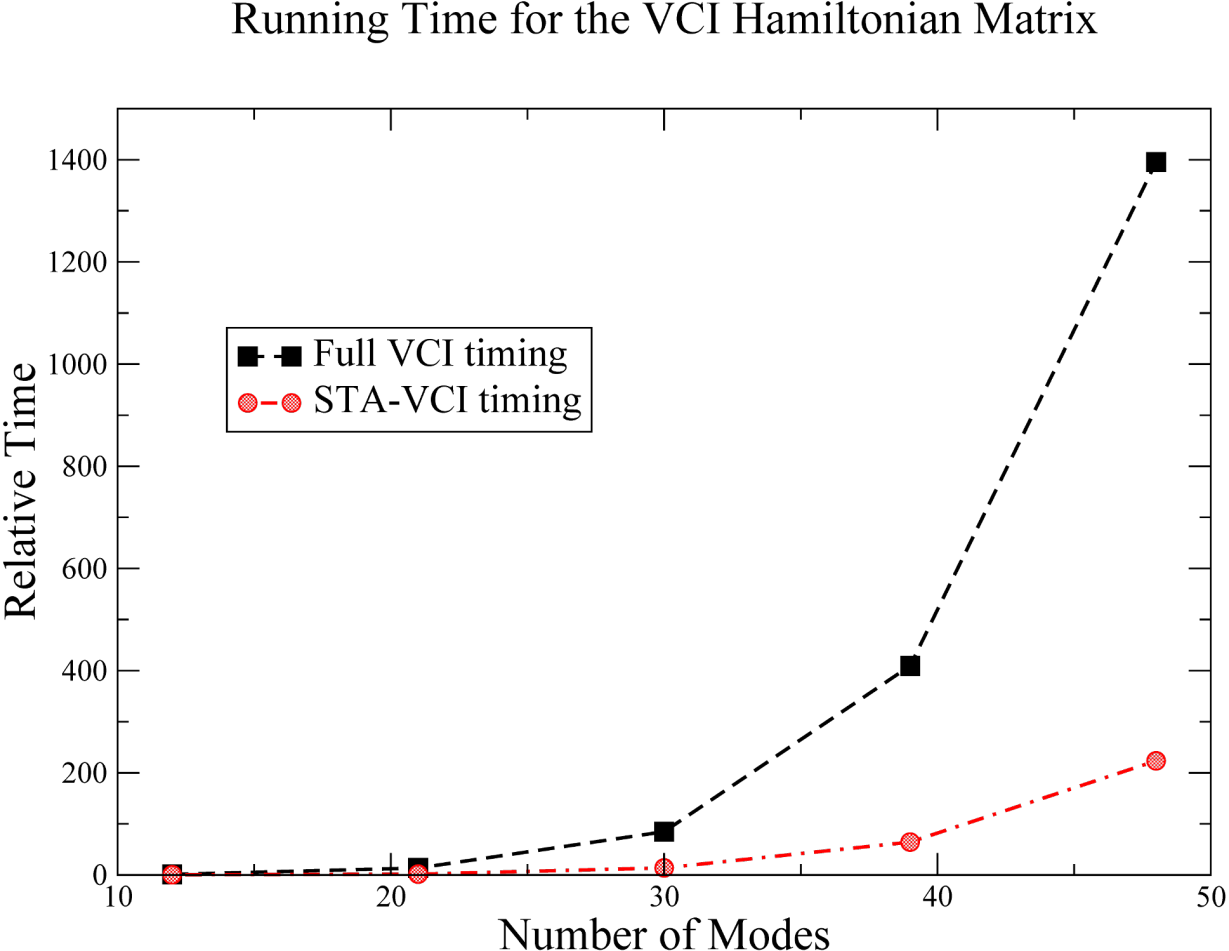
Representative timing for the construction of the Hamiltonian VCI matrix for a series of aliphatic alcohols (methanol to pentanol), for both standard VCI (Full VCI) and STA–VCI methods. All timings are relative to methanol using the standard VCI method.

This method improves the relative timing of calculations of the vibrational configuration interactions, since the computation of the VCI matrix elements are much less demanding. This type of speed-up is shown in Figure 4.

**Figure 5:**
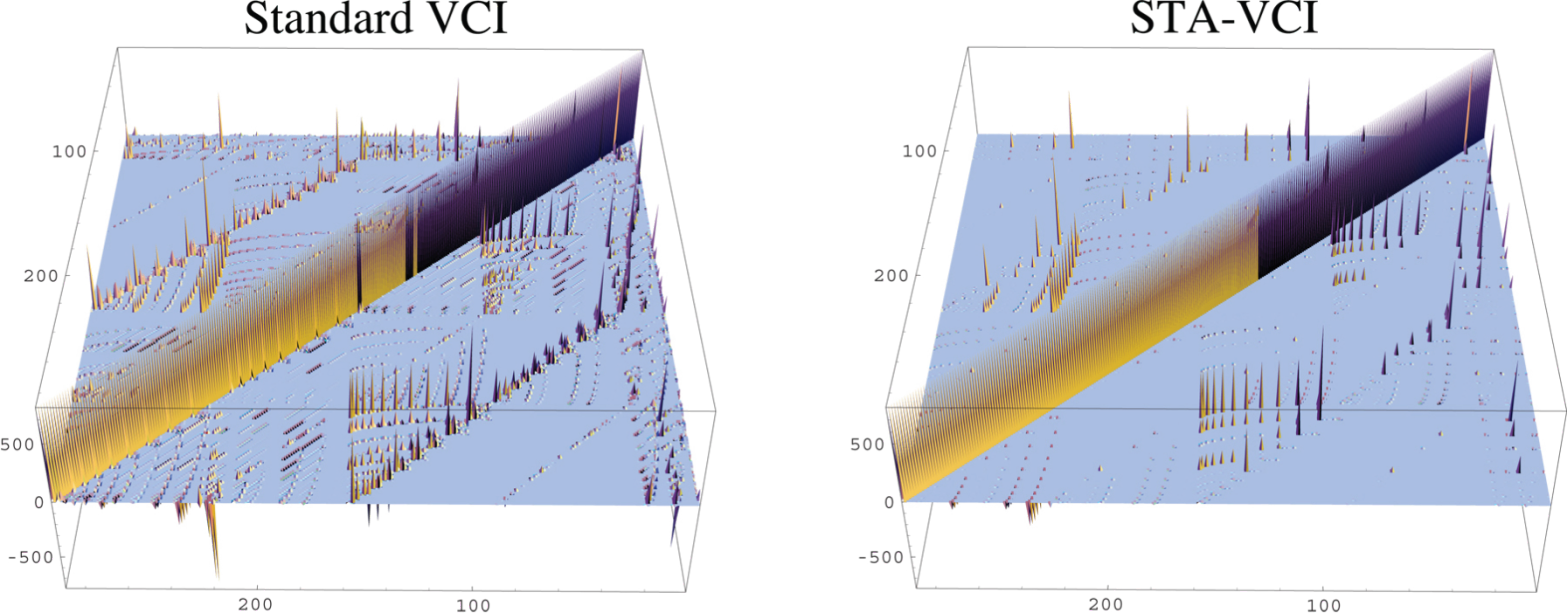
Graphical representation of the VCI matrix elements for a standard VCI and a STA–VCI calculation on methanol using the same basis. For the STA–VCI method, the OH-stretch mode is coupled to all other modes (*N_Ã_* = 1). Note the difference in the occurrence of off-diagonal elements between standard VCI and STA–VCI.

Moreover, our approach generates a sparser VCI matrix representation (Figure 5) that is better adapted to the Davidson algorithm. The STA–VCI approach allows the computation of anharmonic frequencies with a low computational cost for the potential energy surface since only a subset of the intermodal coupling is computed.

#### Applications

**Figure 6:**
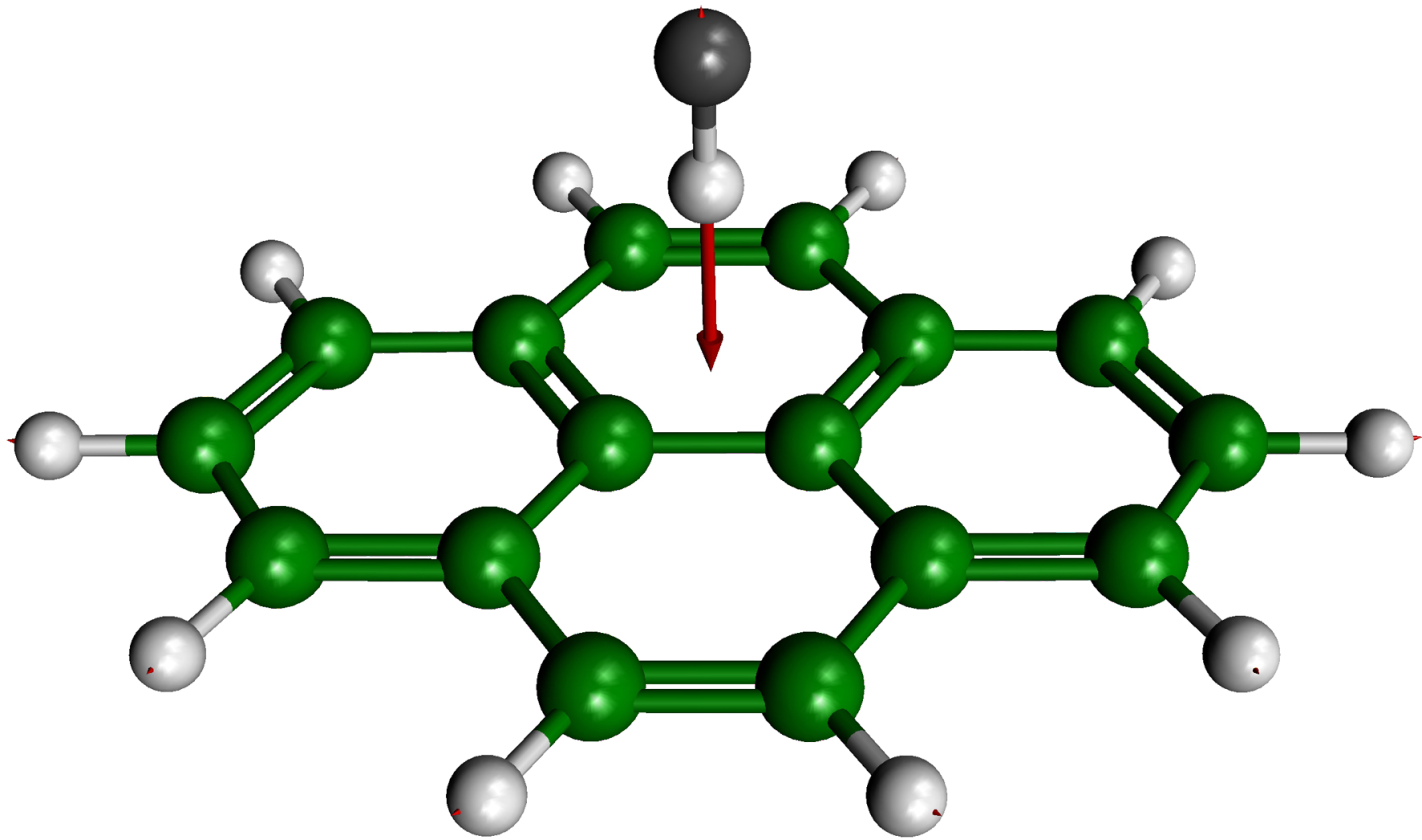
Hydrogen fluoride adsorbed on a pyrene molecule. The arrow represents the HF stretching mode.

The methods presented above allow us to treat large molecular systems and have been used to compute the OH-stretch frequency of benzoic acid (system with 39 modes) [40], for example. Our method can also be applied to larger systems and the STA approach was used to compute the anharmonic stretch frequency of hydrogen fluoride adsorbed on pyrene (Figure 6), a model system for the adsorption of HF on graphite.

The potential energy surface (mode–mode representation) is computed at a moderate ab initio cost (MP2/SBK level of theory). This system is challenging as it contains 78 modes and the weak π–hydrogen bond requires correlated electronic structure methods. The computed harmonic frequency for the stretching mode of HF adsorbed on pyrene is 3661 cm^−1^ which is −112 cm^−1^ away from that of the free HF molecule, ω(HF) = 3773 cm^−1^ at this level of theory. At the VSCF/VCI level of vibrational theory and using the STA approach we obtain 3477 cm^−1^ which corresponds to a shift of −135 cm^−1^ from the gas-phase molecule stretch frequency, ν(HF) = 3612 cm^−1^ from the 1-D vibrational solutions. To the best of our knowledge the HF-stretch frequency of hydrogen fluoride on pyrene has not been measured, however the spectrum of HF–benzene (a much smaller, yet similar system) has been measured by Andrews et al. [41]. The observed HF-stretch frequency is 3795 cm^−1^ in an argon matrix, which corresponds to a shift of −166 cm^−1^ compared to the vibrational stretch of free HF in a gas phase as reported by Herzberg [42] [ν(HF) = 3961 cm^−1^].

We see that both harmonic and anharmonic calculations show the correct trend, i.e., a red shift of the HF frequency caused by the weak interaction with an aromatic compound. We note, however, that including anharmonicity causes almost a 20% change compared to the harmonic value and that the anharmonic results are in closer agreement with experiment (19% deviation), albeit performed on a related system. By comparing the experimental, free, HF-stretch frequency to our anharmonic results, it is obvious that the curvature of the MP2/SBK PES is much too shallow. Nevertheless, we are mainly interested in the effect of anharmonicity on the vibrational frequencies of adsorbed systems and thus focus on the vibrational shift, where this systematic error can be expected to compensate. Interestingly, scaling the vibrational harmonic frequencies – as is commonly done in a number of studies – does not improve much the prediction of the frequency shift at the harmonic level. Indeed, if we use the experimental HF-stretch frequency to compute a scaling factor [ν(HF,exp)/ω(HF) = 1.050], we obtain only a very modest improvement of the shift value (−118 cm^−1^) which is still 30% away from the experiment. This can be compared to the anharmonic prediction of the shift, which does not use any scaling factor. Thus, we note that, for a PES computed at the same level of ab initio theory, the predictive power of the harmonic approximation for adsorption-induced vibrational shifts remains rather limited compared to an anharmonic calculation. This is mainly due to the nature of the harmonic model, that is the assumption of a quadratic shape of the PES at the minimum, which is inadequate for adsorbed molecules as their energetics can be markedly affected by the presence of a surface.

### Iterative perturbative screening of the configuration space

When we consider large molecules, the main drawback of variational calculations is the rapid growth in the number of basis functions necessary in the VCI space. As the size of the molecule increases, the number of normal modes increases and so does the number of possible virtual excitations that can be performed to generate the VCI basis. Thus, even with an efficient VCI implementation, both the computational effort and the storage requirements grow rapidly for large systems, which severely limits the application of the VSCF/VCI scheme to large systems. Such a drawback is not confined to our implementation; in general, the principal difficulty in the development of any given variational method suitable for large molecular systems is the exponential growth in the size of the vibrational basis with the number of atoms.

We therefore implemented a different kind of VCI approach that overcomes these issues, and adapted the variation–perturbation method proposed by Pouchan and Zaki [43] to our STA–VSCF/VCI code. This method was originally proposed by Malrieu and co-workers for electronic structure theory [44], and later inspired a number of vibrational studies (see Brodersen and Lolck [45], for example). The implementation of Pouchan et al. uses a virtual basis set based on a product of harmonic oscillator wave functions and thus the number of configurations necessary to cover the active space is quite large for extended anharmonic systems. Our implementation uses VSCF step as a method to efficiently reduce the size of the configuration space.

The main idea of this approach is based on the separation of the full virtual configurational basis set 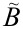 into:


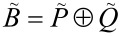


where 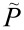 is the active configuration subspace used to perform VCI calculations, and 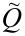 is the subspace that contains virtual configurations, which do not contribute much to the true VCI eigenstate. This is based on the observation that, for most VCI calculations, the converged solutions usually only use about 1% of the relatively large virtual excitation basis set. This implies that there are a number of “preferred” excitations for a particular vibrational state and that the remaining excitations have a very limited impact on the solution. We use a perturbative preselection of the virtual states in order to capture the essential excitations, followed by a variational VCI in the preselected excitation space. The method then uses the new VCI solution as a reference to perform a new screening of the excitations and adds the relevant ones to the VCI space accordingly. This iterative multi-reference perturbative selection technique (VCIPSI) manages to keep the variational space manageably small (well below 2000 configurations in practice) and practically removes the VCI stage from the observed scaling.

Once the convergence of the 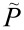 space is reached, we suggested that the effect of the remaining neglected configurations left over in the 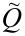 space can be easily accounted for by perturbation theory. We add a MP2 perturbative correction to the last eigenstate energies obtained by diagonalisation of the Hamiltonian in the converged active space 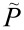. This perturbative correction, computed with the converged non-active space 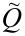 and the final eigenvalue, is called VCIPSI–PT2.

This new approach, combined with the STA technique in order to reduce the number of underlying ab initio calculations needed to compute the PES, provides a dramatic time saving and a much reduced memory usage compared to traditional approaches. This method had enabled us to compute the OH-stretch frequency of benzoic acid nearly 10 times faster than the standard VCI approach, with comparable accuracy [46]. Our results show that there is excellent agreement between the vibrational frequency computed using the STA–VSCF/VCI approach and the STA–VCIPSI–PT2 technique. The new VCIPSI algorithm reduces the memory footprint by a factor close to 200 for the STA–VCIPSI–PT2 method compared to VCI. Moreover, the total time taken by the VCIPSI calculation is reduced by a factor of seven compared to standard VSCF/VCI. These two observations demonstrate the efficiency of the STA–VCIPSI method for the treatment of molecular systems with a large number of normal modes.

Our implementation is very scalable and allows the investigation of larger systems such as an adsorbed 4-mercaptopyridine molecule on an Au(111) surface, using the partial Hessian method described in more details in the next section. The results obtained are in a good agreement with experiment [4] and allow the identification of possible adsorption sites for 4-mercaptopyridine using vibrational data alone, thus leading to a new type of structure determination for adsorbed organic molecules.

### Description of adsorbate vibrations

Before performing an anharmonic calculation for adsorbed molecules on a surface, we need to determine the normal mode vectors of the system. This can be a computationally demanding task for large systems, and the normal mode analysis of adsorbates can be efficiently carried out using the partial Hessian technique [47] instead. Here, the Hessian matrix is split into two parts, one part is calculated explicitly while the matrix elements of the other part are set to zero. The full 3*N_a_*-dimensional space is divided into an “active” and an “inactive” subspace. One set of atoms is allowed to move while the other set is kept frozen during the Hessian calculation. The Hessian matrix, **H**, is organised such that the active and the inactive atoms each form one block on the diagonal, and the off-diagonal elements contain the coupling terms between active and inactive atoms:

[11]
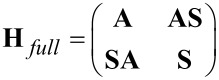


where sub-matrix **A** contains only the force constants of the active part of the system (fragment *A*), usually made up of the adsorbate but which might also include a few surface atoms. Sub-matrix **S** in Equation 11 is usually set to zero to account for the inactive substrate (remaining atoms of the surface, fragment *S*). Sub-matrix **A** is then the only matrix needing diagonalisation, yielding 3*N_A_* normal modes and frequencies, where *N_A_* is the number of active atoms.

The partial Hessian technique reduces the number of dimensions of the system and focuses on a part of interest. It is justified only if fragment *S* is heavy compared to fragment *A*, which is the case in surface calculations where fragment *A* contains mostly the adsorbate atoms and *S* a large proportion of surface atoms; or more generally, if the modes examined are localised in one part of the system. In general, the eigenvectors and eigenvalues of the partial Hessian will converge towards the eigenvectors and eigenvalues of the full Hessian as the number of inactive atoms decreases (with an increasing coupling between the two fragments included in the Hessian).

**Figure 7:**
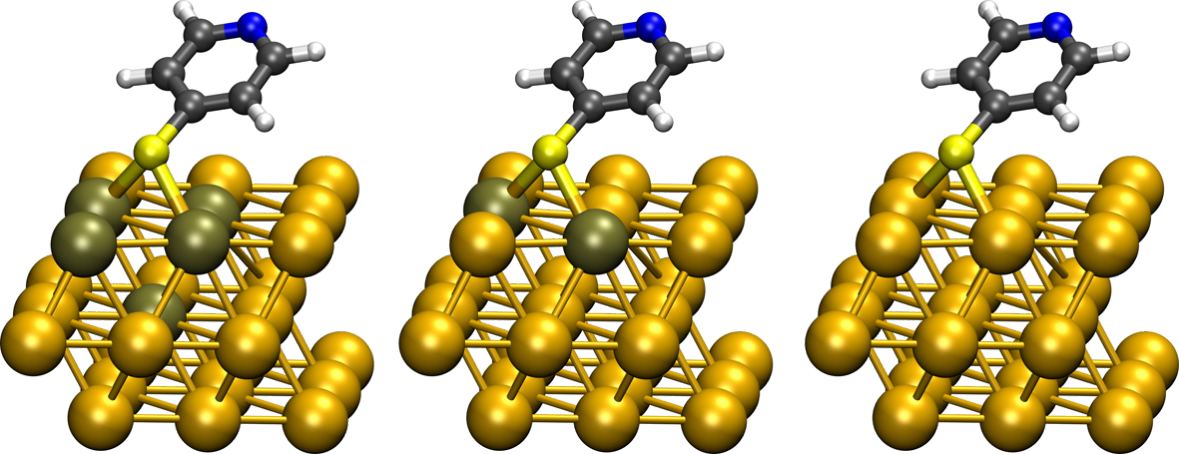
Active atoms for the partial Hessian: 4-Mercaptopyridine adsorbate plus different number of Au atoms, indicated by a darker color; **left**: mpy-ads-5, **middle**: mpy-ads-2, **right**: mpy-ads-0.

We will illustrate this rapid convergence behaviour for the harmonic frequencies of 4-mercaptopyridine (mpy) adsorbed on Au(111) (Figure 7) for different sizes of the partial Hessian matrix. The largest active system, mpy-ads-5, contains five gold atoms (the two gold atoms directly bonded to the sulfur atom, and the gold atoms which are bonded to both the first two gold atoms), thus yielding 48 normal modes. The next size down, mpy-ads-2, contains two gold atoms, both connected to the sulfur atom, giving 39 normal modes. Finally, mpy-ads-0 is a minimal model that does not include any gold atoms in the partial Hessian matrix (33 modes).

The calculations were carried out using the Vasp [48] program and projector augmented wave (PAW) pseudo potentials [49] optimized for the Perdew–Burke–Ernzerhof (PBE) [50] exchange-correlation functional. A 3 × 3 surface unit cell was used, with a vacuum layer ≈17.5 Å thick, and three layers of Au atoms. The plane-wave cutoff was set to 400 eV, and the cutoff for the augmentation charges to 450 eV. The Brillouin zone was sampled using 25 k-points.

The harmonic frequencies, ω*_i_*, obtained for each model are shown in Table 2. We observe that the three models are virtually identical for all modes down to ω = 395 cm^−1^. Two of the Hessians were chosen to contain Au atoms in order to enable a possible coupling between surface modes and the adsorbate, while in mpy-ads-0 this coupling is excluded from the start. From the point of view of the definition of a partial Hessian, the smaller mpy-ads-0 model is better justified, as the active fragment is much lighter than the inactive fragment. However, since adsorbate modes are usually localised on the adsorbate rather than on the surface, the inclusion of Au atoms in the active region should only have a limited effect. This is also supported by Table 2: Only the low frequencies of mpy-ads-5 and mpy-ads-0 differ by more than a wavenumber, thus showing that the high-frequency adsorbate modes converge very quickly with the number of active atoms. However, one should be careful when choosing the computationally cheaper mpy-ads-0 model for an anharmonic treatment of molecule–surface vibrations. Indeed, this reduced-dimension model does not contain any degrees of freedom on the surface atoms and therefore no vibrational coupling is possible between the adsorbate atoms and the surface. This is particularly important for surfaces made of lighter atoms, e.g., carbon-based or silicon-based materials, where the coupling between adsorbate and surface is expected to be much larger. Note that in this case, the main assumption of the partial Hessian formalism (effectively that the surface has an infinite mass) is no longer guaranteed and rigorous convergence tests should be performed.

**Table 2:** Harmonic frequencies for mpy-ads-5, mpy-ads-2 and mpy-ads-0 (PBE/PAW); in parentheses deviation of mpy-ads-2 and mpy-ads-0 with respect to mpy-ads-5; in cm^−1^. Mode numbers from mpy-ads-5. Assignment of normal mode vibrations: ν – stretching, δ – in-plane bending, γ – out-of-plane bending, ρ – rocking, χ – wagging, τ – twisting.

mode no.	mpy-ads-5	mpy-ads-2	mpy-ads-0	normal mode

48	3121	3121 (0)	3121 (0)	ν(CH)
47	3121	3121 (0)	3121 (0)	ν(CH)
46	3084	3084 (0)	3084 (0)	ν(CH)
45	3081	3081 (0)	3081 (0)	ν(CH)
44	1544	1544 (0)	1544 (0)	δ(HCC,HCN), ν(CC)
43	1532	1532 (0)	1532 (0)	δ(HCC,CCC), ν(CC,CN)
42	1451	1451 (0)	1451 (0)	δ(HCC,HCN), ν(CC,CN)
41	1389	1389 (0)	1389 (0)	δ(HCC,HCN), ν(CC)
40	1305	1305 (0)	1305 (0)	δ(HCC,HCN,SCC)
39	1274	1274 (0)	1274 (0)	δ(HCC,SCC), ν(CN)
38	1211	1211 (0)	1211 (0)	δ(HCC,HCN,CCC)
37	1074	1074 (0)	1074 (0)	δ(HCC,CCC,CNC), ν(SC)
36	1073	1073 (0)	1073 (0)	δ(HCC,CCC)
35	1058	1058 (0)	1058 (0)	δ(CCC,CNC), ν(SC)
34	972	972 (0)	972 (0)	δ(CCC,CNC)
33	961	961 (0)	961 (0)	τ(HCC)
32	944	944 (0)	944 (0)	τ(HCC)
31	838	838 (0)	838 (0)	χ(HCC)
30	787	787 (0)	787 (0)	χ(HCC)
29	720	720 (0)	720 (0)	χ(CCC,CNC)
28	679	679 (0)	679 (0)	δ(CCC,CNC), ν(SC)
27	652	652 (0)	652 (0)	δ(CCC,SCC)
26	484	484 (0)	484 (0)	γ(SCC)
25	395	395 (0)	394 (−1)	δ(AuSC), ring pulsation
24	357	357 (0)	357 (0)	ρ(HCC,CNC)
23	304	304 (0)	303 (−1)	*δ*(AuSAu,AuSC,SCC)
22	234	234 (0)	228 (−6)	δ(AuSC), ν(AuS), ring deformation
21	150	150 (0)	140 (−10)	δ(AuSC), ring deformation
20	129	129 (0)	117 (−12)	δ(AuSAu)
19	96	—	—	Au lattice
18	88	—	—	Au lattice
17	87	—	—	Au lattice
16	87	—	—	Au lattice
15	81	—	—	Au lattice
14	80	—	—	Au lattice
13	76	78 (2)	64 (−12)	frustrated rotation
12	72	—	—	Au lattice
11	70	70 (0)	—	Au lattice
10	66	69 (3)	—	Au lattice
9	65	65 (0)	—	Au lattice
8	61	63 (2)	—	Au lattice
7	60	—	—	Au lattice
6	56	53 (3)	—	Au lattice
5	50	52 (2)	—	ring pulsation, ν(CC)
4	46	—	—	Au lattice
3	33	33 (0)	33 (0)	frustrated rotation
2	24	24 (0)	25 (1)	frustrated translation
1	17	18 (1)	19 (2)	frustrated rotation

### Scalable generation of potential energy surfaces

The main issue is to find a PES representation that is scalable and computationally manageable for large systems on the order of 50 atoms or more. Given that the number of degrees of freedom increases sharply with the size of the system, it is necessary to follow a systematic approach. Such an approach enables an automatic construction of the PES and provides a way of increasing PES accuracy by including higher order *n*-body terms in the potential expansion. In general, the 1-D terms are the largest contributors to the PES, followed by the 2-D terms that add couplings between the degrees of freedom. These coupling terms typically contribute significantly less than the 1-D terms but have important implications for vibrational resonances and energy transfer. The 3-D and higher terms add smaller corrections to the PES that are only required for a very high accuracy description. Note that the presence in the PES of terms greater than second order invalidates the direct isomorphism with electronic structure theory and often requires a different methodology.

The computational bottleneck of the direct-VSCF/VMP/VCI methods is the generation of accurate PES. With the fast algorithms introduced earlier, it is possible to significantly reduce the computational demands by taking into account only strong couplings. In the next section we discuss two PES generation schemes that both use delocalised computational resources. The first one is more suited to small scale calculations on a single computer cluster, while the second approach enables PVSCF to take advantage of multiple clusters at remote locations through grid computing.

#### Computational task farming

The potential energy surface required for the direct-VSCF calculation is calculated on a grid of points. The atoms are displaced along the normal mode vectors in steps whose size depends on the vibrational frequency of the mode considered. The total energy is then calculated at each of these *n*_grid_ grid points. Thus, a large number of single-point total energy (SPE) calculations are necessary for the construction of the PES. All of these SPE calculations are performed by an electronic structure program and are independent of each other and can thus be computed simultaneously using many processors. Our calculations were run on the bwGRiD [51] cluster at the university of Ulm, Germany, with unix scripts handling the job submission. All SPE calculations are submitted to the queueing system in bundles of maximum-job-number calculations, so that the queueing system handles the task distribution. On the bwGRiD cluster, each node has eight processors, such that eight calculations are performed in each job.

Without the automatisation and parallelisation of the PES construction, direct-VSCF calculations for interfacial systems of the size mentioned earlier would be computationally inaccessible. For example, the 4-mpy/Au(111) system with adsorbate at a bridge position contains 39 normal modes (if the Au atoms which are directly connected to the sulfur atom are included in the partial Hessian analysis). For this PES, *n*_grid_ = 16,624 SPE calculations are necessary for the 1-D PES, and 189,696 SPE calculations are required for the 2-D PES (39 × 38/2 mode–mode couplings, each of them using 16 × 16 grid points). The computational effort for the parallel calculation is no longer so heavily dependent on the system size, but more on the computation time for one SPE calculation.

#### Grid interface

Even after restricting the PES to include only the strong couplings, for a large system the number of SPE calculations remains quite large, usually between 10^3^ and 10^6^. The same problem also occurs during construction of the Hessian matrix: The number of displacements, which can be estimated as 6*n*_active_(3*n*_active_ + 1), grows very rapidly with the number of active atoms, *n*_active_. In both cases, the amount of processing time (wall time) can be reduced through parallel computing, the obvious technique being to distribute serial tasks across available computing cores. This method can be easily implemented and yields directly to a linear scaling approach, however, in order to use computational resources efficiently, some important questions need to be considered.

First, some queueing systems on grid resources distribute jobs on each node, and not to each core, such that we need to start several serial jobs on each node simultaneously, in order to optimise computational power use. Unfortunately, the different jobs, which run on the same node, may take different computational time. This situation happens very often, because during the PES generation the displacements far from the equilibrium position usually require a longer iteration cycle to achieve convergence. It is also very hard to predict how many nodes and cores may be expected, due to different priority policies for each grid location. Thus, we need a balancing mechanism, which allows us to load all cores on all available nodes evenly. The solution involves implementing a distribution system that submits the grid points dynamically to each location. Instead of submitting a batch script, we submit a special universal executable script (UES) that connects to an SQL database, gets the next free grid point, related files and appropriate external executable script (EES), and runs EES on the particular node and core. This additional EES provides a way to extend functionality dynamically and to run a few different jobs using several ab initio programs simultaneously. Because we want to be able to run jobs on different clusters, grids and individual nodes with different kinds of processors and under various operating systems, all scripts have to be cross-platform. In our implementation of UES, we have chosen the perl language, as it has pure MySQL and PostgreSQL database interfaces that do not use dynamic libraries written in other languages. This is a critical point given that usually MySQL/pSQL client programs and libraries are not installed on the computational nodes. Moreover, perl provides a command line option to specify the locations of non-standard modules, which makes the installation procedure simple and flexible.

Second, due to the unreliable nature of distributed computing across various locations, some results can go missing for various reasons. In order to avoid missing points, we need an intelligent system to recognise and react to various failures, such as when a node goes down, job killed by queueing system or abnormally terminated due to convergence problems, network or SQL server troubles, and so on. Such a system was implemented in two stages: After the calculation of a grid point, the EES parses the output file and looks for results. It then sends the results to UES and returns an exit code. Depending on this code, the results will be uploaded back into the database, or the current point or even all points within a project may be marked as erroneous and will be no longer considered. Moreover, if there are no results during a specified period of time, it is assumed that something went wrong and the grid point will be resubmitted.

**Figure 8:**
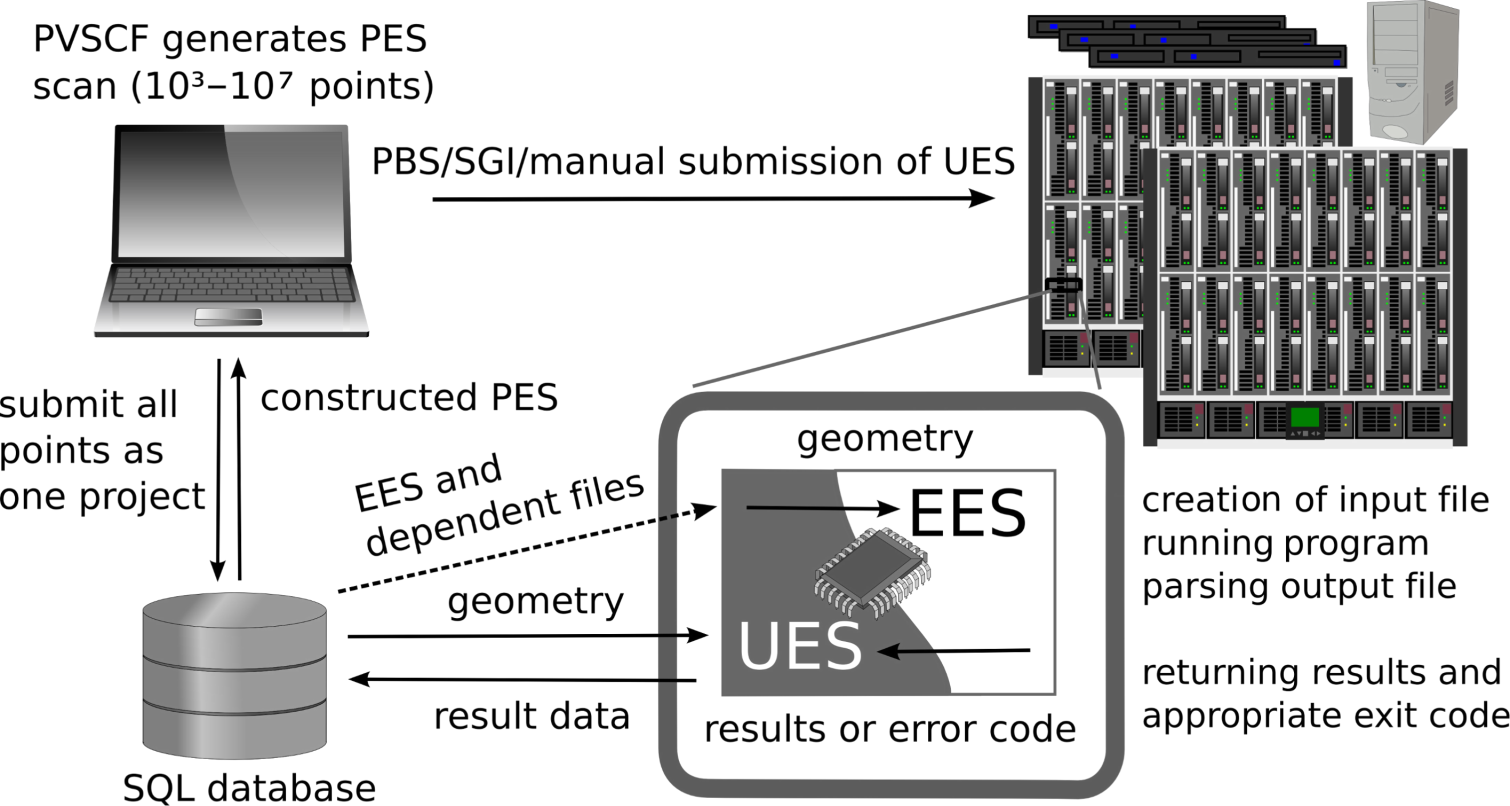
Diagrammatic representation of the grid interface.

In summary, the grid-based PES construction process is composed of the following steps (Figure 8):

1. Generate the set of grid points and upload them to the database. Upload the EES designed specifically to perform the total energy calculation, along with dependent files, such as templates, external basis sets, restart files, etc.
2. Submit batch scripts using PBS, SGI or any other batch system, which will start one UES for each CPU, and/or start UES manually on the local workstation.
3. After UES is started, it downloads the EES and corresponding files once for each grid location, then gets the first available grid point, runs EES and provides it with the geometry of the system at the selected grid point. The EES reads the geometries, produces a valid input file from a template file, runs the particular ab initio program, parses the results and sends it back to UES or returns an appropriate error code. After termination of EES, UES returns results to the database or marks the grid point as defective and tries to download the geometry of the next grid point. This step is repeated until no more points are available.
4. Retrieving constructed PES and the list of non-converged points from the database.

### Periodic density functional theory for molecular PES

In this section, we examine the accuracy provided by periodic density functional codes and their suitability for the construction of PES for anharmonic calculations. The use of periodic DFT codes is desirable for two reasons: A treatment of periodicity is a necessity for the correct description of molecule–metal interfaces, and periodic DFT codes are usually more efficient than standard molecular codes for large systems. Our group [4] and others [52] have shown that, provided the potential energy surface satisfies the periodicity conditions, the Γ-approximation provides an adequate description of the vibrations in periodic systems. If we focus mainly on the vibrational spectrum of adsorbed molecules, the description of surface phonons (which would require a periodic vibrational approach) is of lesser interest and a Γ-point representation leads to a realistic calculation. Moreover, in-phase vibrations (i.e., at the Γ-point) usually correspond to the most intense transitions observed in linear optical spectroscopy. We focus here on the thiophene molecule as it is a sizeable system of 21 vibrational modes (or 210 mode–mode couplings), and it has been shown to form self-assembled monolayers on gold surfaces [53] and as such is a system of relevance to surface science. This molecule is big enough to be a chemically meaningful system, but also small enough that it can be used as a benchmark system to compare DFT with ab initio calculations.

The optimal geometry of thiophene in the gas phase was obtained using Møller–Plesset perturbation theory (MP2) with TZVPP basis set (GAMESS-US [22]) and density functional theory (DFT) with the various functionals and TZVPP (GAMESS-US) or MOLOPT-TZV2P basis sets (CP2K [54]). For the CP2K calculations, the cell size is 10 × 10 × 14 Å^3^ and the periodic solver is used with a 400 eV density cutoff. Pseudo potentials are used for all atoms and are optimised for the respective functionals (e.g., GTH-PBE for PBE), except for sulfur for which the GTH-BLYP pseudo potential was used instead of GTH-HCTH120, due to the lack of GTH-HCTH120 parameters for the sulfur atom. The GTH-BLYP pseudo potential was selected as it gives the best optimal structure.

The calculated geometrical parameters are compared to experimental ones in Table 3. According to the root-mean-square (RMS) deviation values for distances and angles, the PBE and HCTH120 functionals (CP2K) give the best overall agreement. Our ab initio reference method, MP2/TZVPP, gives results of similar quality to PBE.

**Table 3:** Calculated and experimental molecular geometry of thiophene in the gas phase.

	DFT (GAMESS-US)			DFT (CP2K)						
				
mode	B3LYP	HCTH120		BLYP	BP86	PBE	HCTH120		MP2	Exp. [57]

r(C–S), Å	1.728	1.723		1.726	1.722	1.712	1.714		1.717	1.714(0)
r(C=C), Å	1.364	1.369		1.375	1.374	1.376	1.375		1.377	1.369(6)
r(C–C), Å	1.424	1.421		1.427	1.424	1.424	1.422		1.415	1.424(3)
r(=C–H), Å	1.077	1.079		1.081	1.084	1.085	1.082		1.075	1.077(6)
r(–C–H), Å	1.080	1.083		1.084	1.084	1.088	1.084		1.078	1.080(5)
*α*(CSC), °	91.6	91.9		91.8	92.1	92.2	92.3		92.1	92.1(7)
*α*(SCC), °	111.5	111.4		111.3	111.4	111.4	111.4		111.4	111(.47)
*α*(CCC), °	112.7	112.7		112.7	112.6	112.5	112.5		112.6	112(.45)
*α*(SCH), °	120.1	120.0		120.1	120.1	120.0	120.0		120.6	119(.85)
*α*(CCH), °	124.0	124.1		123.9	124.1	124.2	124.1		124.4	124.2(7)

RMSD, 10^−3^ Å	6.8	4.5		6.4	5.2	5.6	3.6		5.7	0.0
RMSD, °	0.32	0.20		0.29	0.16	0.08	0.12		0.35	0.00

The calculated anharmonic vibrational frequencies of thiophene in the gas phase are shown in Table 4, with PES computed using each of these three levels of electronic structure theory. We use the assignment of fundamental transitions by Rico and coworkers [55]. The vibrational calculations are performed using VSCF/VCI in curvilinear coordinates [12] on an equidistant grid with 16 points. We allow up to seven excitation quanta in the VCI basis. In spite of the fact that PBE gives a very good result for the geometry of thiophene, its performance for the prediction of anharmonic frequencies is very poor for this molecule. This is particularly evident for the stretching frequencies and seems to indicate that the atoms are too weakly bound with this functional. A similar behaviour was also observed by Handy and coworkers [56]. In contrast to PBE, the HCTH120 anharmonic frequencies slightly underestimate the experimental values but overall show a very good agreement with the experimental data. The situation is slightly worse at the MP2/TZVPP level due mainly to an overestimation of the strength of single bond stretching.

**Table 4:** Anharmonic frequencies of the fundamental vibrational modes of thiophene in the gas phase (in cm^−1^). The mode assignment convention is: ν – stretching, δ – bending, τ – twisting. The subscript “a” denotes an antisymmetric mode. The CH-stretching and CH-bending modes are labelled according to their irreducible symmetry representation in *C*_2_*_ν_*.

	DFT					DFT			
				
mode	PBE	HCTH120	MP2	Exp. [58]	mode	PBE	HCTH120	MP2	Exp. [58]

τ(C=C–S–C)	410	451	459	452	δ(CH)*a*_1_	1016	1071	1094	1082
τ(C=C–C=C)	521	552	575	564	δ(CH)*b*_2_	1017	1088	1095	1085
δ(CSC)	554	608	612	609	δ(CH)*b*_2_	1174	1239	1266	1256
	637	652	676	683	δ(CH)*a*_1_	1285	1351	1346	1364
	677	694	727	712	ν(C–C)	1329	1422	1427	1410
ν_a_(SC)	693	760	755	754	ν_a_(C–C)	1422	1502	1485	1510
ν(ring)	777	828	849	840	ν(CH)*b*_2_	2877	3038	3122	3087
ν(SC)	805	846	854	866	ν(CH)*a*_1_	2931	3060	3141	3097
δ(ring) + ν_a_(SC)	807	864	879	873	ν(CH)*b*_2_	2919	3103	3174	3125
	839	875	893	900	ν(CH)*a*_1_	2972	3103	3178	3126
	982	1040	1047	1036					
					*RMSD*	99	20	23	0

Our results show that a good agreement between predicted and experimental geometry does not guarantee a reliable prediction of the anharmonic vibrational frequencies. The PBE functional is very popular in surface science and solid state physics but we show that its description of the cohesion between atoms can be an issue for the description of molecular vibrations. While MP2-based calculation of the PES has been shown in the past to lead to reliable anharmonic frequencies for molecular systems [59], perturbation theory remains computationally expensive for periodic systems. We observe that HCTH120 seems to be a much cheaper alternative to MP2 periodic systems, and that it provides a better description of the vibrational properties than the ubiquitous PBE functional. However, the HCTH family of functionals do not satisfy the uniform electron gas limit and were mainly parameterised for molecular systems [60].

### Assessing metal–metal bonds through anharmonic calculations

The description of binding forces in metals is still a topical issue, where density functional theory has so far been very successful. Indeed, the complexity of transition metal bonding, potentially involving a number of degenerate electronic states, appears to be easily described by gradient-corrected functionals such as PBE. In order to further investigate the suitability of some currently used density functionals for the description of gold–gold interactions, we compare our results for the geometries and binding energies of the Au_2_–Au_10_ clusters to the reported theoretical and experimental results from other authors. We also give a brief outlook of our study of the harmonic and anharmonic vibrational frequencies for these gold clusters, as vibrational properties are directly connected to the curvature of the potential energy landscape and thus provide important information on the overall quality of the PES.

In order to obtain local minimum energy structures, we use the Nelder and Mead version of the Simplex method [61–62], together with five different empirical models: The Murrell–Mottram potential [63] with the parameters used by Wilson and Johnston [64], the Sutton–Chen potential [65], the Gupta potential [66] with the parametrisation defined by Cleri and Rosato [67], the Glue model as developed by Ercolessi et al. [68], and the Voter–Chen version of the embedded atom model (EAM) [69–71]. In order to reproduce planar structures using these potentials, geometrical constraints are introduced. Planar structures have already been predicted by high level theory to be the global minima for the smallest clusters. The size of planar-to-nonplanar transition in gold clusters varies between *n* = 7 and *n* = 15, depending on the DFT approach used. Our DFT calculations are carried out using a plane-wave basis set with relativistic ultra-soft Vanderbilt (VDB) pseudo potentials [72]. We perform plane-wave DFT calculations using the CPMD code (version 3.11.1) [73]. We use the Perdew–Burke–Ernzerhof PBE [50] functional and other functionals such as BP86 [74–75], BLYP [74,76] and LDA [77]. We use periodic boundary conditions and a cubic supercell of (15 Å)^3^ to avoid strong interactions between neighbouring clusters and a plane-wave energy cutoff of 30 Ry (408 eV). Additionally, we optimise a selected set of structures up to Au_7_, using the MP2 method. These calculations are carried out using the SBKJC(1*f*) basis set [78–79]. Geometry optimisations were performed using GAMESS-US code [22], but only on a selected set of structures. In contrast to most DFT calculations, in which planar structures are predicted as the global minimum even for sizes above *n* = 10, MP2 predicts the first non-planar minimum energy structure at a size of *n* = 7 [78,80].

**Figure 9:**
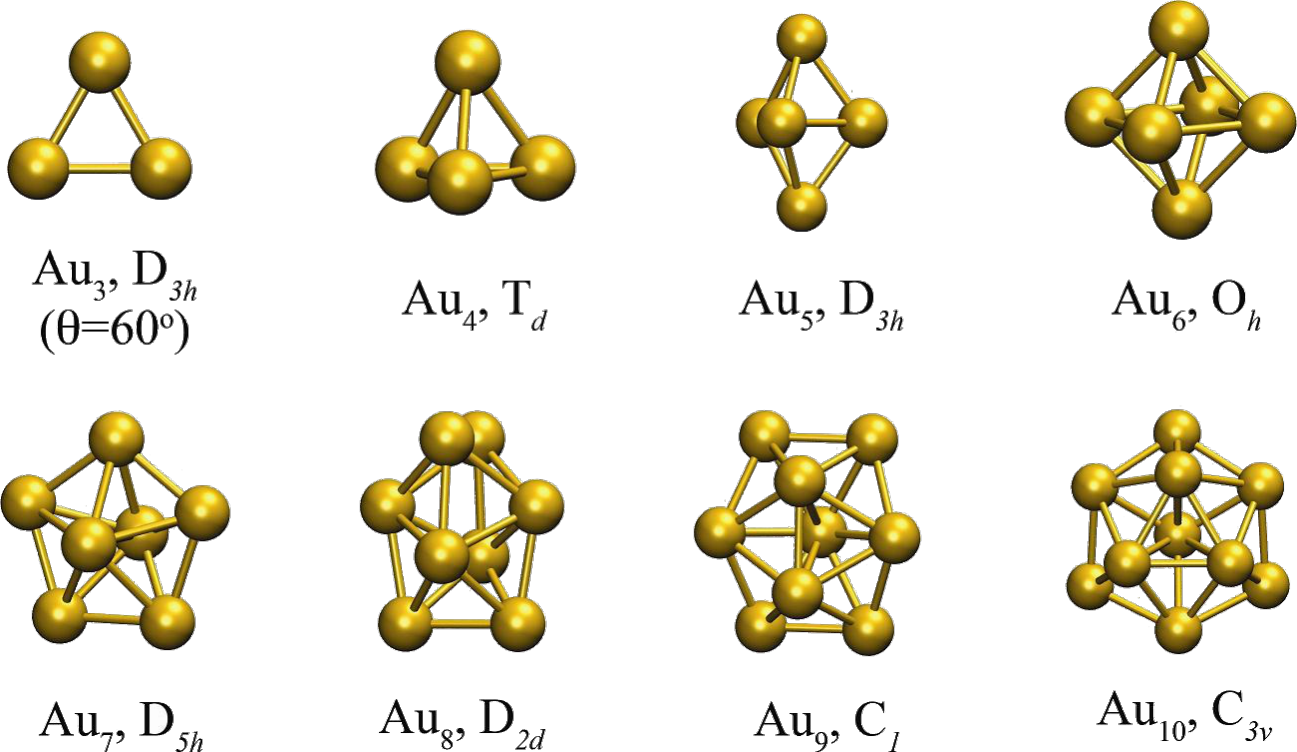
Global minimum energy structures for Au_3_–Au_10_ clusters obtained for empirical potentials, along with their symmetry point group.

**Figure 10:**
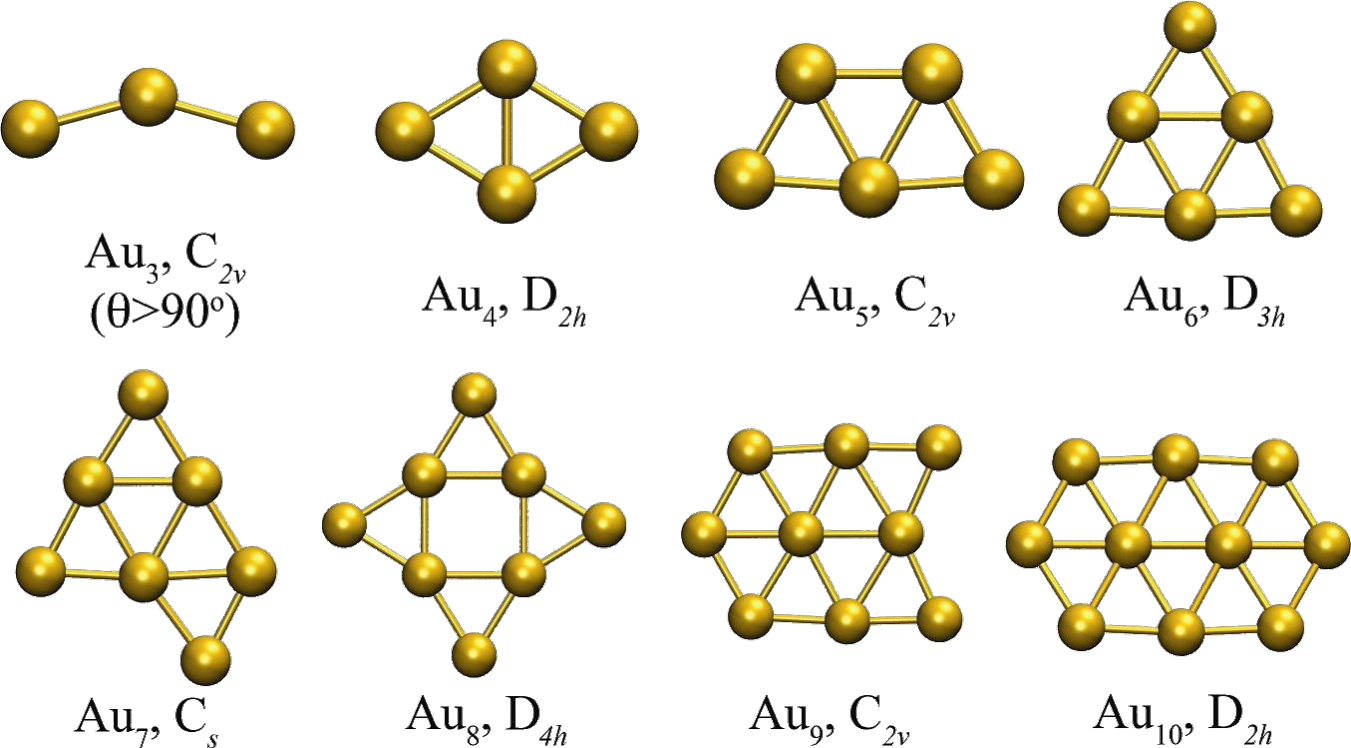
Global minimum energy structures for Au_3_–Au_10_ clusters obtained for PBE/VDB, along with their symmetry point group. Structures with equivalent but not identical geometry can be obtained from the empirical potentials by including constraints.

We use our fast-VSCF/VCI [11,40] technique to compute the anharmonic frequencies of the lowest DFT energy minima for each size. The global minimum energy structures obtained with the empirical potentials are shown in Figure 9. All of them are non-planar except for the trivial cases, Au_2_ and Au_3_. The global minimum energy structures obtained using PBE/VDB, which are all planar, are shown in Figure 10.

**Figure 11:**
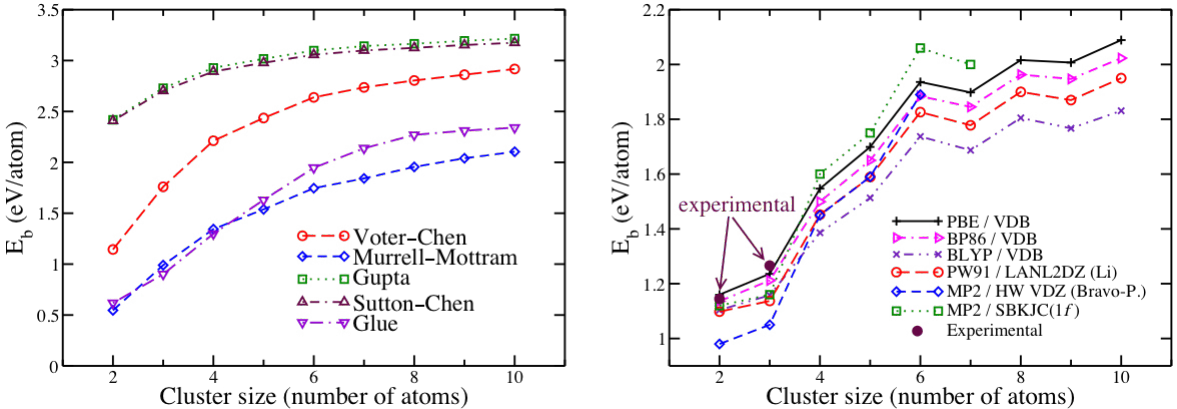
Left: Binding energies calculated for Au_2_–Au_10_ clusters using different empirical potentials. The values correspond to the global minimum energy structures, which are always non-planar (except for Au_2_ and Au_3_). Right: Binding energies obtained using DFT and MP2 calculations, compared to DFT values reported by Li [81], to MP2 values reported by Bravo-Perez [82], and to experimental values reported by Bishea and Morse [83–84]. All structures are planar (MP2 global minimum energy structure for Au_7_ is non-planar, but here we show the value corresponding to the planar structure). For Au_3_, the structure reported by Bravo–Perez is the equilateral triangle.

The binding energies for the non-planar empirical global minimum structures are shown in Figure 11 (left). Among the empirical potentials studied, the experimental binding energy of the gold dimer is best reproduced by the Voter–Chen potential. This empirical potential also provides the best prediction for the bond length and is also the most suitable potential to reproduce the features of the high-level PBE/VDB potential energy surfaces. It is therefore used to pre-scan PES in the fast-VSCF method. The right part of Figure 11 shows a plot of the binding energies for the various DFT approaches and MP2/SBKJC(1*f*), compared to energy values reported in other studies and to available experimental values.

We find that PBE/VDB reproduces best the experimental binding energies of Au_2_ and Au_3_, as well as the bond length of Au_2_. Experimental binding energies for larger clusters have not been reported. The calculated energies using PBE/VDB are in close agreement with the values reported by Li et al. [81] using the PW91 functional [85] with a LANL2DZ basis (~0.1 eV on average). Better agreement is found with binding energies reported by Xiao et al. [86], who used the PW91 functional with the projector augmented wave (PAW) method (differences of ~0.01 eV/atom). We assume that, for each cluster size, the characteristics of the vibrational spectrum are determined only by the global minimum energy structure. This assumption is valid for small clusters but, as size increases, the presence of near degenerate minima is likely to give rise to extra transitions due to other isomers. The Voter–Chen version of the embedded-atom model is used to prescan the PES for the fast-VSCF/VCI calculations.

For the gold dimer, the computed harmonic frequency using PBE/VDB is ω = 176.2 cm^−1^, and the anharmonic frequency is ν = 175.3 cm^−1^. These values are ~8% lower than the experimental frequencies, ω = 190.9 cm^−1^ and ν = 190.1 cm^−1^, respectively [83]. Nevertheless, the calculated anharmonic frequency is 0.9 cm^−1^ lower than the calculated harmonic frequency, almost the same difference as that between the experimental values.

A comparison of the RMS deviation between the harmonic and anharmonic frequencies (Table 5) shows that the anharmonicity for all the small clusters studied is around ~1.0 cm^−1^. Even if gold clusters do not show large overall vibrational anharmonicity, some specific vibrational transitions show large relative anharmonicity. The modes exhibiting larger anharmonicity are generally associated with bending motions of the molecule. We also note that vibrational anharmonicity does not affect the planarity of the Au_7_ cluster.

**Table 5:** Differences between anharmonic (ν) and harmonic (ω) frequencies for the global minima of bare planar clusters Au_2_ to Au_10_ obtained using PBE/VDB, in cm^−1^. The anharmonic results are obtained from fast-VSCF/VCI calculations for clusters with size and from standard VSCF/VCI calculations for the smaller clusters. For each cluster, we report the difference for the highest frequency mode (ν_max_) in the cluster, the maximum difference (MAXD), and the rms deviation for all normal modes (RMSD).

Cluster size	ν – *ω*		
	
*n*	ν*_max_*	MAXD	RMSD

2	−0.9	−0.9	0.9
3	−1.1	1.6	1.2
4	−1.8	−1.8	0.9
5	−1.2	−2.1	1.1
6	−0.8	−1.6	0.6
7	0.8	0.8	0.4
8	0.0	−2.6	0.8
9	−0.7	−2.0	0.6
10	−1.1	−1.2	0.5

## Conclusion

In this paper we have shown that a careful implementation of the direct vibrational self-consistent field method enables us to investigate the quantum vibrational properties of extended systems. Our physically intuitive picture of “preferential” communication channels between normal modes provides a fast and accurate way of performing these calculations, at a fraction of the computational cost of the standard approaches. This new perspective opens the door to novel ways of obtaining accurate anharmonic spectra directly from ab initio or density functional theory data, thus making a direct link between theory and experiment. We believe that the techniques we have developed lay the foundations for a rigorous description of the vibrational spectra of complex systems beyond the harmonic approximation, and provide a very promising tool for future investigations of vibrational, bound states in large systems. The applications presented enabled us to assess the quality of the standard electronic structure techniques against experimental results and revealed the strengths and weaknesses of certain types of exchange and correlation functionals. Setting such a benchmark provides a way of systematically improving and cross-checking the description of inter-atomic interactions, which is a necessary building block for the theoretical description of nanostructures.
